# SARS-CoV-2 surveillance in a hospital and control of an outbreak on a geriatric ward using whole genome sequencing

**DOI:** 10.1016/j.infpip.2024.100383

**Published:** 2024-07-06

**Authors:** Hanno Schmidt, Niels Lemmermann, Matthias Linke, Sven-Ernö Bikár, Stefan Runkel, Susann Schweiger-Seemann, Susanne Gerber, André Michel, Thomas Hankeln, Marina Veith, Wolfgang Kohnen, Bodo Plachter

**Affiliations:** a SARS-CoV-2 Sequencing Consortium, University Medical Center of the Johannes Gutenberg-University Mainz, Mainz, Germany; b Institute of Virology, University Medical Center of the Johannes Gutenberg-University Mainz, Mainz, Germany; c Institute of Virology, Rheinische Friedrich Wilhelm University of Bonn, Bonn, Germany; d Institute of Human Genetics, University Medical Center of the Johannes Gutenberg-University Mainz, Mainz, Germany; e StarSEQ GmbH, Mainz, Germany; f Transfusion Centre, University Medical Center of the Johannes Gutenberg-University Mainz, Mainz, Germany; g Health Care Department, University Medical Center of the Johannes Gutenberg-University Mainz, Mainz, Germany; h Institute of Organismal and Molecular Evolutionary Biology, Johannes Gutenberg-University Mainz, Mainz, Germany; i Center for General Medicine and Geriatrics, University Medical Center of the Johannes Gutenberg-University Mainz, Mainz, Germany; j Department of Hygiene and Infection Prevention, University Medical Center of the Johannes Gutenberg-University Mainz, Mainz, Germany

**Keywords:** SARS-CoV-2, Whole genome sequencing, Variant monitoring, Covid-19, Hygiene management

## Abstract

**Background:**

During the SARS-CoV-2 pandemic, dominant viral variants were repeatedly replaced by new variants with altered properties, frequently changing the dynamics of the infection event, as well as the effectiveness of vaccines and therapeutics. SARS-CoV-2 variant monitoring by whole genome sequencing was established at the University Medical Center Mainz, Germany to support patient management during the pandemic.

**Methods:**

SARS-CoV-2 RNA samples from the University Medical Center were analysed weekly with whole genome sequencing. The genome sequences obtained were aligned with sequences from public databases to perform variant assignment. For classification purposes, phylogenetic trees were constructed to map the variant distribution in the clinical settings and the current outbreak events at that time. We describe the surveillance procedures using an example from a geriatric ward.

**Results:**

For monitoring, a time series was created covering two years of the pandemic. The changes from the Alpha to the Delta and the Omicron variants of SARS-CoV-2 could thus be precisely observed. The increasingly rapid switch of Omicron subvariants in the recent past could be tracked. The elucidation of phylogenetic relationships between circulating strains allowed conclusions about transmission pathways. Using an example from a geriatric ward, we demonstrated how variant monitoring by whole genome sequencing supported the infection prevention and control procedures on a ward and contribute to the control of outbreaks.

**Conclusions:**

This example of SARS-CoV-2 demonstrates the effectiveness of targeted, local monitoring by molecular variant analysis. The program proved to be instrumental in controlling an outbreak on a geriatric ward.

## Introduction

The Covid-19 pandemic caused by the by SARS-CoV-2 virus caused nearly 800 million infections and has claimed about seven million lives worldwide [1]. The virus is constantly changing and new variants have repeatedly emerged, some of which showing markedly different characteristics in terms of disease progression and the effectiveness of vaccines and antiviral therapeutics [2]. Early variants, such as Delta, showed higher pathogenicity [3], whereas the current variant Omicron is associated with higher infectivity and lower pathogenicity [4]. The protective effect of available vaccines has declined with the emergence of Omicron [5], leading to efforts to produce variant-adapted products [6]. In addition, some viral lineages developed resistance to monoclonal antibodies [7] or antiviral drugs such as remdesivir [8]. These variant-specific properties thus fundamentally shift transmission kinetics and treatment options. Therefore, knowledge of the variants circulating in a hospital and other healthcare settings is of great practical relevance. In addition, evaluation of the variants enables the tracing of viral transmission routes on individual wards and between wards. By tracking the variants circulating on wards it is possible to distinguish between transmissions within the ward that require close attention [9,10], and external introductions that were incorrectly considered as such at first glance [11,12]. This approach may save resources and allow better targeted infection prevention and control resources. A systematic review of implementations of whole genome sequencing-based variant identification in healthcare settings found that 90% of published efforts supported hypotheses for nosocomial transmissions of SARS-CoV-2 [13]. Findings such as these are informative for making adjustments to infection prevention and control procedures to prevent transmission. These often include changes to personal protective equipment practice [14] and social distancing requirements [15], but are situation-specific and therefore vary from case to case [13].

During the pandemic, newly admitted patients and medical staff members at the University Medical Center in Mainz, Germany (UM-Mainz) were routinely tested for SARS-CoV-2 infection by quantitative polymerase chain reaction (qPCR) assays. Positive samples were subsequently subjected to whole genome sequencing. Polymorphisms were analysed and used to identify the underlying specific variant.

Patterns of variant distribution can indicate whether cases within and between individual wards are related. Phylogenetic calculations can be used to reconstruct the relationships of the strains found in even greater detail. By this approach, multiple transmissions to wards and other clinical areas can be distinguished from internal spread within a ward or other clinical area, and infection prevention and control measures can be adjusted accordingly. Here, we present an example of a case in which variant identification by whole genome sequencing was instrumental in controlling a SARS-CoV-2 outbreak on a geriatric ward.

## Methods

During the study observation period, all patients were tested with a PCR test for SARS-CoV-2 infection on admission to the UM-Mainz. In addition, all patients and staff with symptoms of SARS-CoV-2 infection on wards were tested immediately. In this study, SARS-CoV-2-positive samples from new infections found by these tests were subjected to weekly SARS-CoV-2 whole genome sequencing between March 2021 and April 2023.

Whole genome sequencing was performed as follows. RNA was first isolated on an eMAG platform (bioMérieux, Marcy-l’Étoile, France) from an aliquot of the swab samples that were collected for qPCR. The viral genomes were amplified using the NEBNext SARS-CoV-2 Sequencing Kit (New England Biolabs, Ipswich, MA, USA) and the Illumina COVIDSeq Test (Illumina, Inc., San Diego, CA, USA), respectively, based on the ARTIC V3 primer set [16]. The resulting sequencing libraries were sequenced on Illumina instruments (MiSeq, NextSeq 500, and NextSeq 2000) to at least 50-fold depth. Sequence reads were cleaned from adapter sequences and sequences of poor quality (<15) and inadequate length (<30 nt) were removed. Afterwards, the sequence reads were mapped to the SARS-CoV-2 reference genome (NC_045512.2). Consensus sequences were generated with a minimum depth of 20 (positions with lower coverage were masked by “N”). Variant sites with less than 90% support were displayed by respective IUPAC nomenclature characters. Generated consensus sequences were then assigned to respective pango lineages using the tools pangolin, scorpio, and PUSHER and weekly updated pangolin databases [17,18]. This procedure follows the nCoV-minipipe/CovPipe guidelines [19].

In addition, phylogenetic analyses of relationships between isolates were performed regularly. This was done either in MEGAX v.10.1.8 (ClustalW sequence alignment algorithm) or Geneious Prime v.2023.0.4 (MAFFT sequence alignment algorithm), applying the neighbor joining clustering method. These results, together with information from the patients' wards were then used to search for clusters of infection.

The core element of the strategy was the introduction of a consortium which included a multidisciplinary collaboration between key professionals both from the clinical services of the hospital and from other relevant services and institutions. This enabled time-efficient collaboration between experts with different expertise and roles. Bioinformatically analysed sequencing results were evaluated at the Institute of Virology and entered into the hospital's internal diagnostic system. During the study all wards with patients whose isolates had been sequenced were immediately informed about the results of the sequencing. Direct exchange of information between the Institute of Virology, the Department of Hygiene, and the affected wards took place via telephone and e-mail. If the multidisciplinary evaluation suggested that an internal transmission event was likely, the respective ward was informed and the infection prevention and control measures were reviewed and adjusted by the Department of Hygiene. Direct communication between professionals of different disciplines across institutions facilitated the decision-making process for complex cases.

## Results

**Figure 1:**
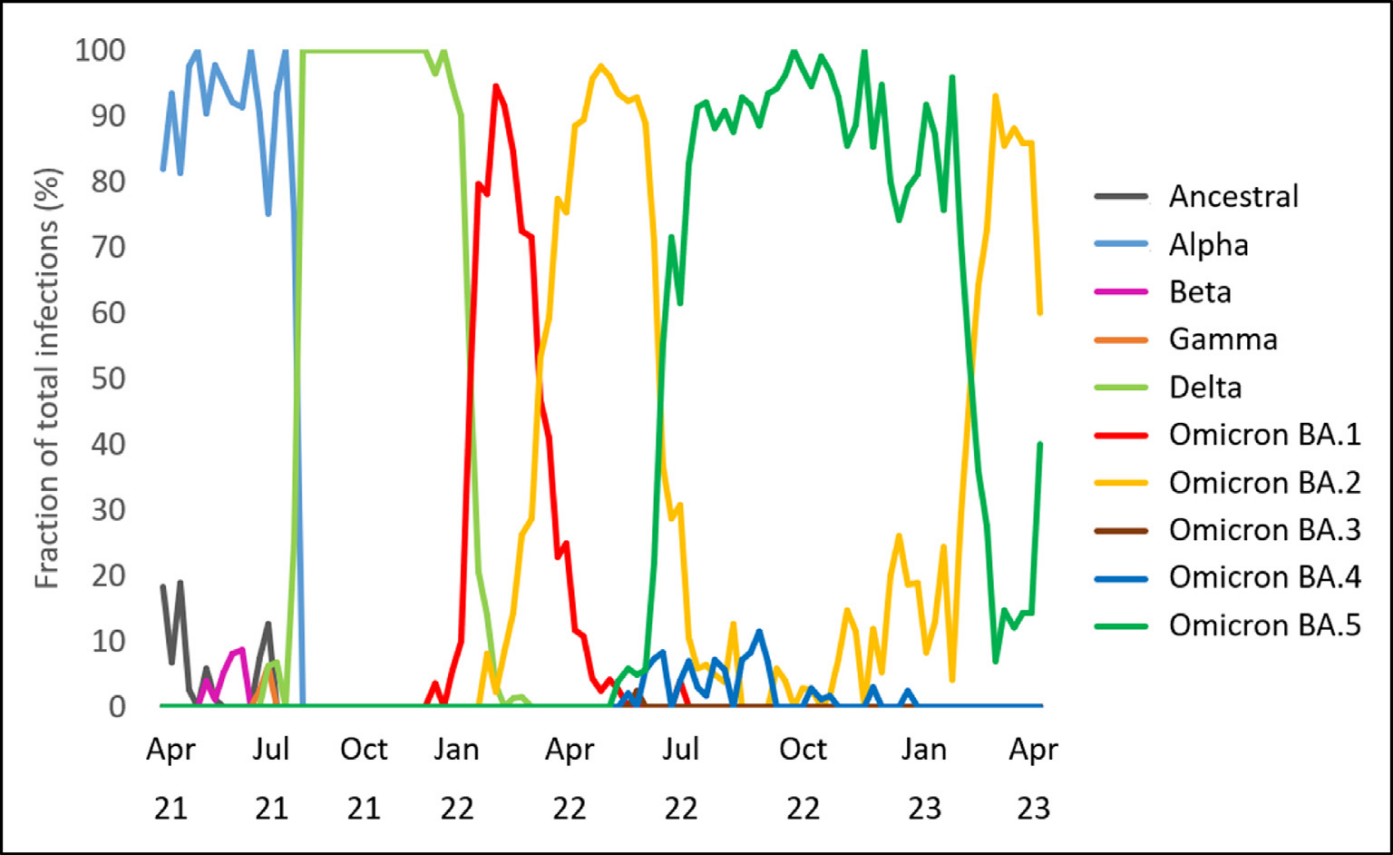
Relative fractions of main lineages in clinical samples. SARS-CoV2-positive samples from March 2021 to April 2023 were sequenced and viruses were subsequently assigned to main lineages of SARS-CoV-2. The relative proportions shown here illustrate the progression of the epidemic and the change between dominant lineages in the clinic. Of the Omicron lineage of the virus that has been dominant since early 2022, the subvariants (BA.1 to BA.5) are shown separately.

In total, around 4,000 samples from UM-Mainz were analysed in the study. The monitoring period from March 2021 to April 2023 covered the occurrence of the Alpha and Delta waves as well as another 15 months in which different Omicron subvariants became dominant (Figure 1). Of note is the multiple rapid replacement of one variant by the next dominant variant. Omicron subvariant BA.2 was the only variant that became the dominant variant again after it had already been almost completely replaced (by BA.5).

**Figure 2:**
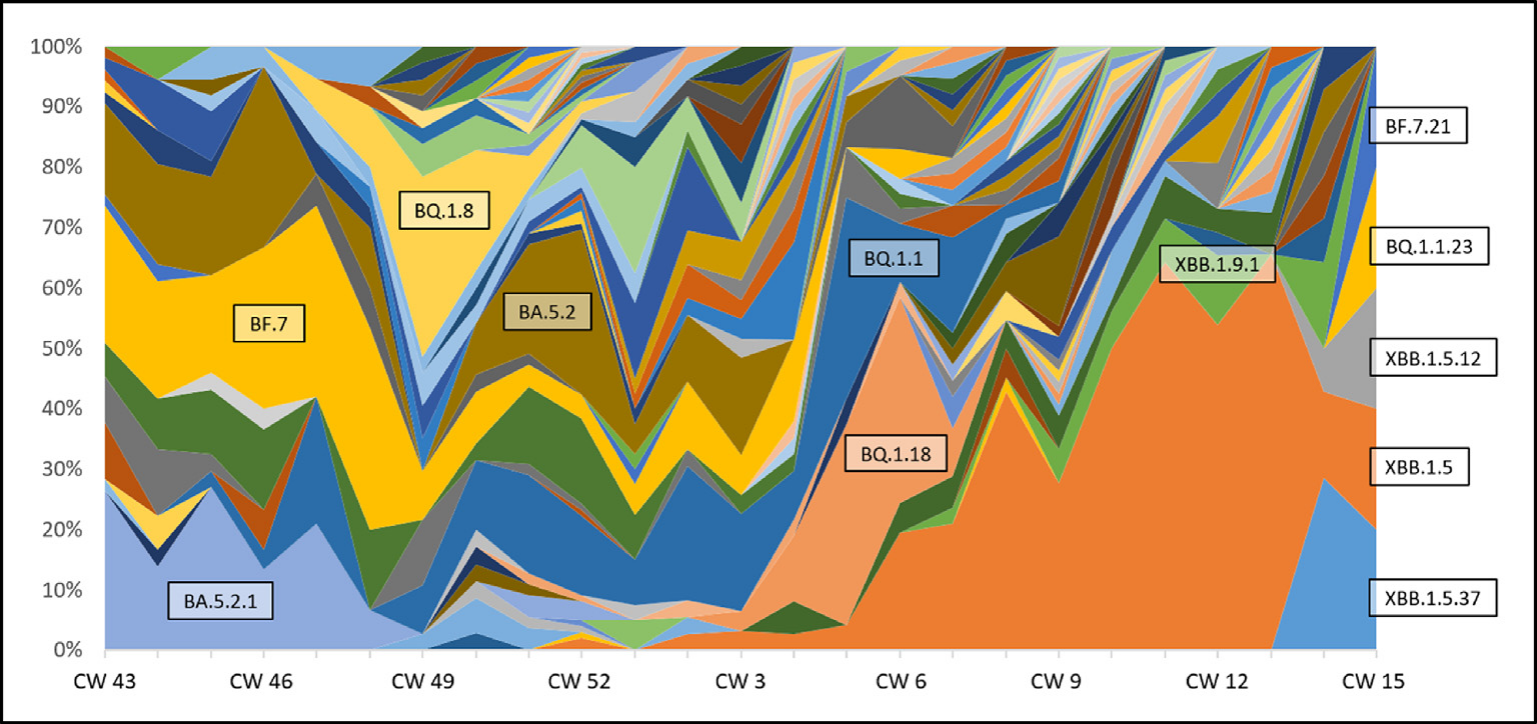
Relative proportions of the subvariants in clinical samples. The detailed breakdown of the variant assignment yielded a highly complex picture. Four months from fall 2022 to spring 2023 are shown. The most frequent variants are labelled in the figure at one of their respective maxima. The appearance of XBB.1.5 in late 2022 (from calendar week 52) and its subsequent sharp increase can clearly be seen. The BQ.1.8 outbreak around week 49 described below can also be seen. CW = calendar week.

The situation became more complex when isolates were divided into subvariants. There were several thousand different variants circulating worldwide, of which often a dozen or more circulated simultaneously in a region. It was not uncommon for these variants to be replaced by new ones again after a few weeks. Without exception, the subvariants belonged to the various Omicron lines in the period under consideration from fall 2022 to spring 2023. BA.5 variants were clearly dominant, only towards the end BA.2 variants appeared more frequently (Figure 2). Of note was variant XBB.1.5, which after initially stable prevalence in the low range (<5% between calendar week 49 in 2022 and calendar week 6 in 2023) became the dominant variant in the clinical areas within a few weeks and achieved a level of dominance not previously observed.

**Figure 3:**
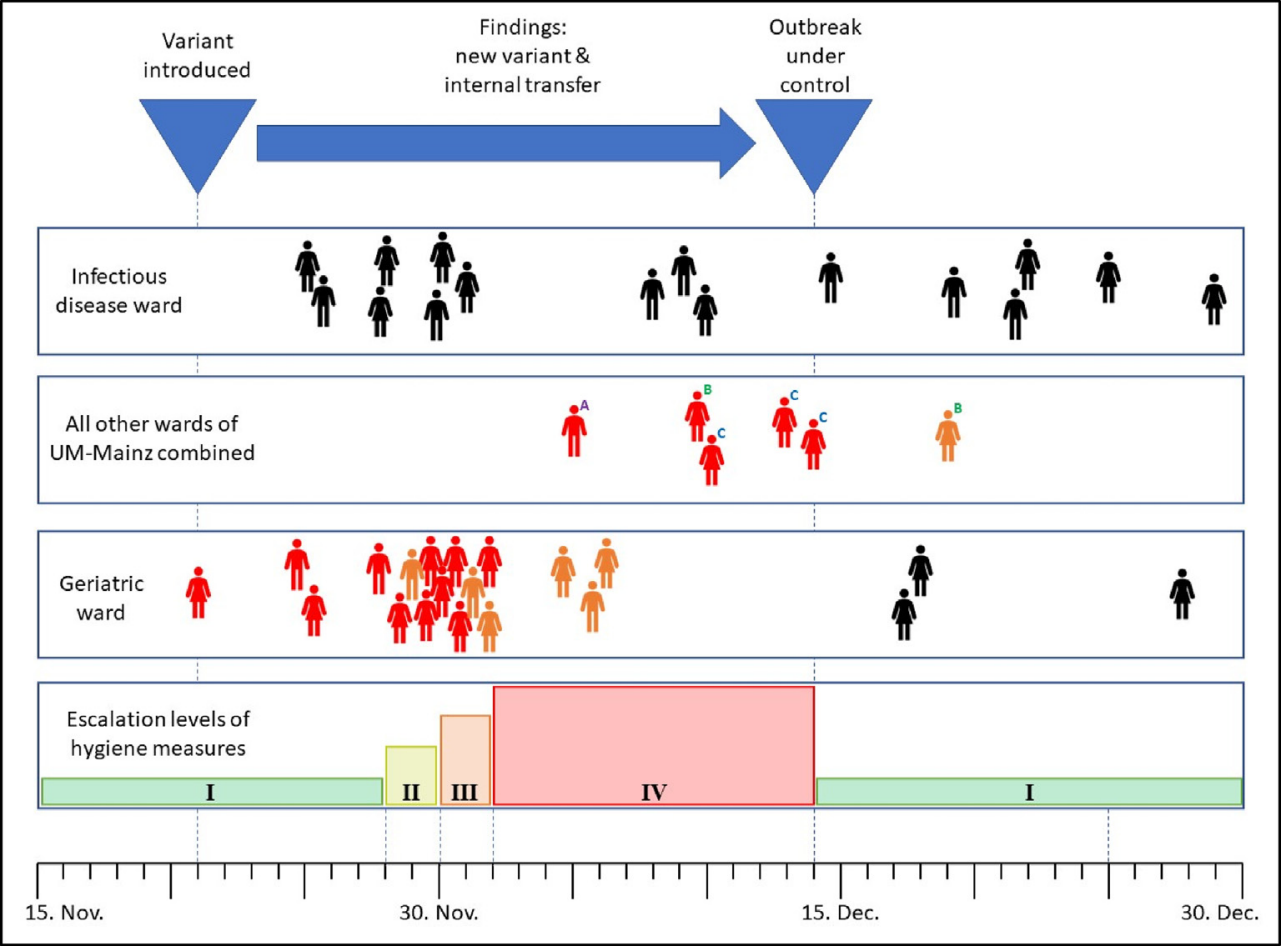
Monitoring and management of a SARS-CoV-2 outbreak on a geriatric ward. The outbreak occurred at the end of 2022. Patients whose SARS-CoV-2 isolate turned out to be BQ.1.8 by sequence analysis are shown in red, medical staff members infected with BQ.1.8 are indicated in orange. All SARS-CoV-2-positive individuals whose isolate was assigned to a different variant by whole genome sequencing are highlighted in black. Shown are cases from the geriatric ward affected by the BQ.1.8 outbreak, cases from an infectious disease ward, caring for COVID-19 patients, and all BQ.1.8 cases from the entire rest of the hospital (non-BQ.1.8 cases are not shown due to their high numbers). A, B, and C are keys for three other wards within UM-Mainz with individual BQ.1.8 cases during the time period shown. No additional BQ.1.8 cases were recorded outside of this time period. As a result of the findings from the whole genome sequencing, hygiene measures were stepwise escalated on the geriatric ward. The escalation levels of hygiene measures were (I, green) standard Covid-19 measures, (II, yellow) zonal segregation to limit internal transmissions on the ward, (III, orange) daily medical staff member screening, (IV, red) isolation of the entire ward with halted patient admission to the ward and a ban on visitors.

In November 2022, a cluster of COVID-19 cases had emerged on a geriatric ward, suggesting that an internal transmission event had occurred. The ward was subdivided into two areas on November 28, 2022 to isolate SARS-CoV-2 infected patients as a first adjustment to the infection prevention and control measures to control transmission (Figure 3). In parallel, isolates from nasopharyngeal swabs of SARS-CoV-2 -positive patients and staff members were sequenced and bioinformatically evaluated. Analysis of the sequence data from the first cases revealed that the isolates were of a variant that had never been detected before in the UM-Mainz. The variant, with the official identifier BQ.1.8, belonged to a new subvariant within the Omicron linage BA.5 and displayed significantly higher infectivity compared with other variants circulating at this time [20]. The high incidence rate of BQ.1.8 was also visible in the overall quantitative monitoring of subvariants at the UM-Mainz (Figure 2). Based on these findings, infection prevention and control measures on the geriatric ward were again escalated (11/30/22) and daily staff testing became mandatory. During these days, the ward recorded additional COVID-19 cases that were also attributable to BQ.1.8 according to genome sequencing. The detection of ongoing transmission within the ward associated with a SARS-CoV-2 variant with high infectivity led to a further escalation of the infection prevention and control measures. These comprised a lockdown of the ward, including stopping patient admissions to the ward and a ban on visitors (12/02/22).

No significant transmission of BQ.1.8 infections to other wards of the UM-Mainz was observed. On one infectious disease ward, which treated large numbers of SARS-CoV-2-positive patients in the pandemic, not a single BQ.1.8 case was identified during the entire study period. From all other clinical departments of the UM-Mainz, a total of six cases were attributed to the BQ.1.8 variant.

**Figure 4:**
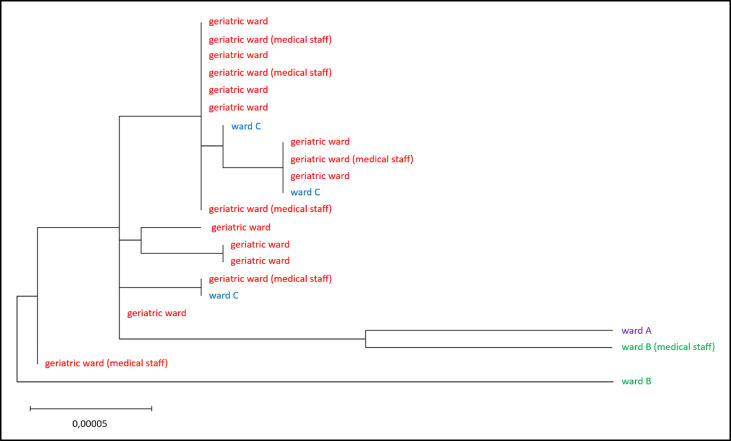
Phylogenetic relationships between the isolates. The relationships between the virus isolates, shown as a phylogenetic tree. All BQ.1.8 isolates identified by whole genome sequencing are included, named after the ward from which they originated, and colored accordingly. The tree topology is indicative of the order of relatedness between samples (qualitative characteristic), and the branch length is indicative of the evolutionary distance between samples/groups (quantitative characteristic). The long branches to the three isolates from patients from wards A and B presumably indicate independent infection events. The very close relationship of the isolates from the geriatric ward and ward C suggests that this could be a case of transmission from one ward to the other. The scale in the lower part of the image indicates the number of substitutions per genome position and thus the sequence dissimilarity as branch length.

Phylogenetic analyses were performed to break down the relationship between isolates from the different wards. A phylogenetic tree was constructed based on a total of 22 aligned sequences of the BQ.1.8 isolates. This phylogeny analysis showed a tight grouping as well as very short branches and thus high sequence similarity for all isolates from the geriatric ward(Figure 4). Three cases from another ward (Ward “C" in Figure 3)) were found to fall in between the samples from the geriatric ward, suggesting close relatedness. However, sequences from viruses in three other samples from two other wards (“A" and “B") were allocated to much longer branches and thus displayed substantial sequence differences compared to the samples from geriatrics. Based on these analyses, conclusions about relationships of the SARS-CoV-2 isolates were drawn and by this, extensive transmissions between wards were felt to be highly unlikely.

## Discussion

During the COVID-19 pandemic caused by the SARS-CoV-2 virus, public health services and healthcare services came under considerable pressure worldwide. Many initiatives to improve infection prevention and control measures were initiated. These measures were often successful in temporarily and regionally reducing the spread of the virus [21,22]. In addition to these general measures, the targeted contact tracing of infections was also implemented [23]. In supporting and strengthening the effectiveness of contact tracing and the investigation of transmission events, whole genome sequencing played an increasingly important role in this context [13,24,25]. Transmission events can be detected more objectively through the direct determination of the same virus variants, and the laboratory processes are more scalable than with the personnel-intensive “detective work” of the authorities and intervention teams [26]. Consequently, variant monitoring was established and expanded at many locations, enabling precise retrospective analyses and thus improved planning for future events. For example, whole genome sequencing and patient monitoring allowed detailed reconstruction of a series of internal transmission events in an emergency department during the third wave of the pandemic, resulting in increased PCR testing and the use of HEPA filtration on affected wards [27].

In this study, we describe the COVID-19 monitoring program at UM-Mainz, which provided valuable data on the incidence of COVID-19 infection in the hospital. The concept was based on the approach of combining contact tracing and whole genome sequencing to maintain and increase the benefits of both in a synergistic manner. However, the efficient dovetailing of the two methods was very demanding. It required timely sample processing, prompt flexibility in implementation of changes in infection prevention and control measures, and most importantly direct, constructive, and trusting communication between all the parties involved. A consortium with the involvement of hygiene managers, virologists, genomic experts, and others was established, and regular, direct communication channels were established. The analysis of four thousand samples from UM-Mainz in two years allowed the precise tracking of the dynamics between the different virus variants and enabled timely adaptation of measures.

The pattern of occurrence, increase, decrease and disappearance of variants observed in the UM-Mainz corresponds well with the patterns documented nationally for Germany [28]. However, Figure 1 also shows patterns that deviate from the national ones. Of note was the replacement of the main lineages (Alpha by Delta, Delta by Omicron) which occurred much faster. This is most likely due to the fact that the processes were observed in a clearly defined spatial framework, while the signals from different regions overlap in nationwide observations, resulting in averaged abundances and thus longer replacement periods. This highlights the strength of local monitoring. Whole genome sequencing served as a close-meshed review of the infection events at UM-Mainz, which was important to support maintenance of patient care in the exceptional situation which occurred because of the COVID-19 pandemic.

The example described here of an outbreak with a novel SARS-CoV-2 variant on a geriatric ward demonstrates the value of the analysis of whole genome sequencing analysis for transmission control, compared with standard monitoring based on qPCR or antigen test approaches. In our case, sequence analysis led to the swift detection of the variant BQ.1.8 which was not seen in the UM-Mainz before. The rapid communication of this finding was important as this variant was known for its particularly high transmission rates and required the escalation of infection prevention and control measures. Furthermore, whole genome sequencing was able to demonstrate that all cases on the ward were related and provide evidence that they resulted from internal transmission events within the ward. This insight was the basis for further escalation of infection prevention and control measures on the affected ward. As a consequence, the outbreak was quickly controlled and further spread on the ward or into other wards in the hospital was prevented. After no new infections were found for 12 days in patients and 8 days in staff, infection prevention and control measures were reduced to the standard that was in place before the outbreak occurred. This was decided since the mean incubation period of COVID-19 is approximately 6 days (95% CI 5.0–6.7 days) for the wildtype [29] and approximately 4 days (95% CI 3.8–4.5) for the Omicron variant [30].

The phylogenetic analyses also showed that the three variants found in samples from ward C were closely related to the variants from the geriatric ward. This suggested that transmission from the geriatric ward to ward C had occurred. Because of the increased workload in geriatrics during the pandemic, there was a requirement to enroll supplementary staff from a pool for service. These staff worked on other wards, depending on clinical demand and thus, transmission events from geriatrics to other wards could have occurred. Supplementary staff were tested daily for SARS-CoV-2 infection. Thus, the route of transmission of BQ.1.8 from the geriatric ward to ward C is unclear at this point. But irrespective of this, the sequence-driven escalation of infection prevention and control measures was effective with at most a single transmission outside the geriatric ward. The control of transmission of SARS-CoV-2 on geriatric wards is considered challenging [9].

## Conclusions

Genome analysis by whole genome sequencing proved to be a valuable tool for SARS-CoV-2 variant monitoring during the pandemic. The data shown here support this and provide an example of how whole genome sequencing can be instrumental for the detection of clusters of infections and for the subsequent control of transmission in a hospital setting.

## Ethics approval

The study has been conducted as part of public health control measures. It has been reviewed by the Ethical Board of the Landeskammer Rheinland-Pfalz (Medical Association of the state of Rhineland-Palatinate, Germany) which waived the requirement for informed consent owing to the fact that no patient- or personnel-specific data are contained in this work, and the data has been analyzed and presented anonymously.

## Availability of data and materials

The sequence data generated and analyzed during the current study are available at the GISAID website (https://gisaid.org/) and upon request from the corresponding author HS.

## CRediT author statement

**Hanno Schmidt:** Supervision, Project administration, Investigation, Data Curation, Visualization, Writing - Original Draft, Writing - Review & Editing. **Niels Lemmermann:** Conceptualization, Funding acquisition, Supervision, Data Curation. **Matthias Linke:** Methodology, Investigation. **Sven-Ernö Bikár:** Methodology, Investigation. **Stefan Runkel:** Funding acquisition, Resources. **Susann Schweiger-Seemann:** Conceptualization, Funding acquisition. **Susanne Gerber:** Conceptualization, Funding acquisition. **André Michel:** Conceptualization, Funding acquisition, Resources. **Thomas Hankeln:** Conceptualization, Funding acquisition, Investigation. **Marina Veith:** Data Curation, Resources. **Wolfgang Kohnen:** Conceptualization, Funding acquisition, Investigation, Resources, Writing - Review & Editing. **Bodo Plachter:** Conceptualization, Funding acquisition, Investigation, Resources, Writing - Original Draft, Writing - Review & Editing.

## Funding

The UM-Mainz sequencing consortium and the present study were supported by the Ministry of Science and 10.13039/100018696Health of the State of Rhineland-Palatinate.

## Conflict of interest statement

The authors declare that they have no competing interests.
